# Conjugated Linoleic Acid Supplementation under a High-Fat Diet Modulates Stomach Protein Expression and Intestinal Microbiota in Adult Mice

**DOI:** 10.1371/journal.pone.0125091

**Published:** 2015-04-27

**Authors:** Alice Chaplin, Pilar Parra, Francisca Serra, Andreu Palou

**Affiliations:** Laboratory of Molecular Biology, Nutrition and Biotechnology, University of the Balearic Islands and CIBER de Fisiopatología de la Obesidad y Nutrición (CIBEROBN), Palma de Mallorca, Spain; Faculty of Biology, SPAIN

## Abstract

The gastrointestinal tract constitutes a physiological interface integrating nutrient and microbiota-host metabolism. Conjugated linoleic acids (CLA) have been reported to contribute to decreased body weight and fat accretion. The modulation by dietary CLA of stomach proteins related to energy homeostasis or microbiota may be involved, although this has not been previously analysed. This is examined in the present study, which aims to underline the potential mechanisms of CLA which contribute to body weight regulation. Adult mice were fed either a normal fat (NF, 12% kJ content as fat) or a high-fat (HF, 43% kJ content as fat) diet. In the latter case, half of the animals received daily oral supplementation of CLA. Expression and content of stomach proteins and specific bacterial populations from caecum were analysed. CLA supplementation was associated with an increase in stomach protein expression, and exerted a prebiotic action on both *Bacteroidetes/Prevotella* and *Akkermansia muciniphila*. However, CLA supplementation was not able to override the negative effects of HF diet on *Bifidobacterium* spp., which was decreased in both HF and HF+CLA groups. Our data show that CLA are able to modulate stomach protein expression and exert a prebiotic effect on specific gut bacterial species.

## Introduction

Obesity is currently growing at an epidemic rate, considered a major health threat around the world, and resulting in an increased risk of diabetes mellitus type II, some types of cancer, fatty liver disease, hypertension, cardiovascular disease and increased mortality. Despite large research efforts on the effects of diet, exercise, education, surgery or drug therapies, there is still no long-term solution to efficiently prevent or counteract obesity [1].

Dietary conjugated linoleic acids (CLA) refer to a mixture of geometric and positional isomers of linoleic acid with conjugated double bonds found mainly in ruminant meat and dairy products [2]. Growing research has shown that the isomers *cis*-9, *trans*-11-CLA and *trans*-10, *cis*-12-CLA in particular have a major role in the regulation of body weight and body fat in both animal [3–9] and human [10–13] studies,

The present study was carried out to further characterise the effects of CLA on body weight management, by addressing certain aspects that to our knowledge have not been studied before. The gastrointestinal tract is the largest endocrine of the body and is responsible for the conversion of food into energy, is metabolically highly active and home to trillions of microbes [14, 15]. The stomach is one of the first sites in the gastrointestinal tract that responds to food intake. It synthesises proteins which have an important role in energy balance and have been shown to be modulated by diet [16–21]. Another interesting component is caecum content, which harbours a large amount of bacteria that carry out a number of functions involved in energy regulation, such as the processing of non-digestible polysaccharides, metabolism of proteins, synthesis of vitamins and production of energy [22–25]. Emerging evidence suggests that the gut microbiota may be involved in obesity [26–28], and that high-fat diet in particular could contribute to the modulation of this bacterial community [29–33]. Therefore, the role of food in modifying gut microbiota towards a more beneficial profile is of great interest [34].

Overall, this highlights the importance of the interplay between food and the different components of the gastrointestinal tract. The aim was to study the potential of CLA in the regulation of both stomach protein expression and specific gut bacterial species in obese mice under a HF diet.

## Materials and Methods

### Animals

Male C57BL/6J mice from Charles River (Barcelona, Spain) weighing 21 ± 0.1 g (5 weeks-old) were housed under standard conditions in cages in groups of 4–5 and kept in a 12-h light:dark cycle at 22°C with food and water *ad libitum*. Cages were Makrolon type III (Tecniplast, Biosis Biologic Systems S.L.) and bedding was Ultrasorb fir shavings (Panlab S.L.U). Bedding was changed weekly. After reception, animals were allowed to acclimatize for a week and divided into groups ensuring equal weight average. Food was changed twice a week, and intake and body weight were recorded every three days throughout the experiment [35]. The animal protocol followed in this study was reviewed and approved by the Bioethical Committee of the University of the Balearic Islands (approval 13^th^ February 2006) and University guidelines for the use and care of laboratory animals were strictly followed. All efforts were made to minimize suffering.

Mice were divided into three treatment groups (n = 8). All diets were prepared by Research Diets (Inc, New Brunswick) and presented as pellets to the animals. Detailed composition of these diets can be found in S1 Table. Mice received one of the following diets for 54 days: a standard normal-fat diet (NF), containing 12% kJ content as fat, used as control, or a high-fat diet (HF), which contained 43% kJ content as fat. Diets were based on the standard rodent diet AIN-76A. Therefore, both diets contained equal proportion of protein (20% kJ content) and carbohydrate was used to adjust the energy content. Thus, NF diet contained 40% (w/w) of sucrose and HF 35% (w/w). Then, a daily dose of CLA was given to half of the animals receiving the HF diet. Tonalin (kindly provided by Cognis) was used as the CLA supplement, providing 6 mg of CLA/day (21.4 nmol/isomer/day), given as an oral gavage. Tonalin TG 80, derived from safflower oil, is composed of triacylglycerols containing approximately 80% CLA with a 50:50 ratio of the active CLA isomers *cis*-9, *trans*-11 and *trans*-10, *cis*-12. At the end of the experiment, body weight did not differ between HF and CLA group, whereas body fat was statistically lower in CLA animals. Complete set of data, including weight of animals, body fat (day 40) and estimated food intake have been previously published [35].

### Sacrifice and sample collection

Sacrifice of all animals was carried out within the animal facilities, at the beginning of the light cycle and after 10h of food deprivation. Animals were anaesthetized with an intraperitoneal injection made up of a mixture of xilacine (10 mg/kg body weight) and ketamine (100 mg/kg body weight). Organs and samples of interest were excised and weighed (stomach and caecum). Stomach was opened and the inside was scraped to collect the mucosa. Caecum was also cut open and content collected. All tissues were rinsed with saline containing 0.1% diethyl pyrocarbonate (Sigma, Madrid, Spain) and snap-frozen at -80°C.

### Quantification of gastric leptin and ghrelin

Stomach mucosa was homogenized at 4°C in 1:3 (w/v) PBS (mM: 137 NaCL, 2.7 KCl, 10 phosphate buffer, pH 7.4) and centrifuged at 7000 x *g* for 2min at 4°C. Total protein was determined after 5-fold dilution of the supernatant with PBS using the Bradford method [36]. Gastric leptin was determined in the initial homogenate with a mouse leptin enzyme-linked immunosorbent assay (ELISA) kit (R&D Systems, Minneapolis, MN). Ghrelin determination in stomach was carried out according to [37]. Stomach homogenate was mixed with 10 volumes 1 M acetic acid containing 20 mM HCl, boiled for 20 min and centrifuged at 7000 x *g* for 2 min at 4°C. The supernatant was lyophilized and resuspended in PBS. Ghrelin concentration in stomach was then determined using a mouse ghrelin enzyme immunosorbent assay (EIA) kit (Phoenix Europe, Karlsruhe, Germany).

### RNA isolation, retrotranscription and real-time qPCR

Total RNA extraction from stomach was carried out with Tripure Reagent (Roche Diagnostic Gmbh, Mannheim, Germany) according to the manufacturer’s instructions. Isolated RNA was quantified using NanoDrop ND-1000 spectrophotometer (NanoDrop Technologies Inc., Wilmington, DE, USA) and its integrity was confirmed using agarose gel electrophoresis.

Samples were retrotranscribed and real-time PCR was carried out for the analysis of stomach proteins. Briefly, 0.25 μg of total RNA (in a final volume of 5 μl) were denatured at 65°C for 10 min and then reverse-transcribed to cDNA using MuLV reverse transcriptase (Applied Biosystems, Madrid, Spain) at 20°C for 15 min, 42°C for 30 min and a final step of 5 min at 95°C in a thermal cycler (Applied Biosystems 2720 Thermal Cycler, Madrid, Spain).

Each PCR was performed with diluted cDNA template, forward and reverse primers (5μM each) and Power SYBR Green PCR Master Mix (Applied Biosystems, CA, USA). Primers were designed and obtained from Sigma Aldrich Química SA (Madrid, Spain) and sequences are described in Table 1. Real-time PCR was performed using the Applied Biosystems StepOnePlus Real-Time PCR Systems (Applied Biosystems) with the following template: 10 min at 95°C followed by a total of 42 temperature cycles (15 s at 95°C and 1 min at 60°C). In order to verify the purity of the products amplified, a melting curve was produced after each run according to the manufacturer's instructions. The threshold cycle (Ct) was calculated by the instrument's software (StepOne Software v2.0), and the relative expression of each gene was calculated as a percentage of NF mice using the 2^−ΔΔCt^ method [38]. Beta-actin was used as the reference gene.

**Table 1:** Nucleotide sequences of primers used for qPCR amplification in mouse stomach.

Mouse genes	Forward primer (5’ to 3’)	Reverse primer (3’ to 5’)	Amplicon size (pb)
Beta-actin	tacagcttcaccaccacagc	tctccagggaggaagaggat	120
Leptin	ttgtcaccaggatcaatgaca	gacaaactcagaatggggtgaag	186
Ghrelin	cagaaagcccagcagagaaa	gaagggagcattgaacctga	144
Mboat4	ttgtgaagggaaggtggag	gagagcagggaaaaagagca	115
Retn	ttccttttcttccttgtccctg	ctttttcttcacgaatgtccc	246
Gpr39	ctgctgattggctttgtatgg	cggttggagaggttcgtg	188
Gcg	tctgacgagatgagcacca	tgactggcacgagatgttg	136
Gcgr	gcacccgaaactacatcca	acacgccctctaccagca	231
Sst	accccagactccgtcagtt	agcctcatctcgtcctgct	169
Sstr	catcgtcaacatcgtcaacc	catcctccacaccgtatcct	194

Forward and reverse sequences designed for qPCR amplification in stomach samples of mice.

### Bacterial profiling by qPCR

Total bacterial DNA was extracted from approximately 50 mg of caecal samples using the E.Z.N.A. Stool DNA kit (Omega Biotek, GA, USA). DNA concentration was determined using a NanoDrop ND-1000 spectrophotometer (NanoDrop Technologies Inc., Wilmington, DE, USA) and its integrity confirmed by agarose gel. Assessment of the presence and relative amount of bacterial species was determined by measuring DNA abundance of the 16S rRNA gene sequences by qPCR with the Applied Biosystems StepOnePlus Real-Time PCR System (Applied Biosystems), following previously described protocols [39, 40]. Specific primers for *Clostridium coccoides*, *Clostridium leptum* and *Lactobacillus* spp. (Firmicutes representatives*); Bacteroides/Prevotella* (Bacteroidetes); *Bifidobacterium* spp. (Actinobacteria); *Akkermansia muciniphila* (Verrucomicrobia); *Enterobacteriaceae* (Proteobacteria); and *Total Bacteria* were obtained from Sigma (Madrid, Spain). *Total Bacteria* refers to a broad-range universal primer that recognizes the conserved region of the 16S rRNA encoding gene for a wide range of bacterial species, and was used to normalize the assay to total bacterial DNA. Sequences are described in Table 2. The threshold cycle was calculated using the 2^−ΔΔCt^ method [38], and relative bacterial content and fold change (FC) were calculated (Log_2_2^−ΔΔCt^). Values were normalized with the average of the NF group.

**Table 2:** Sequence of primers used for bacterial profiling in caecum content.

Phylum	Bacterial Species	Forward primer (5’ to 3’)	Reverse primer (3’ to 5’)
Proteobacteria	Enterobacteriaceae	cattgacgttacccgcagaagaagc	ctctacgagactcaagcttgc^[^ ^39^ ^]^
Actinobacteria	Bifidobacterium spp.	cgcgtcyggtgtgaaag	ccccacatccagcatcca^[^ ^39^ ^]^
Firmicutes	Clostridium coccoides	actcctacgggaggcagc	gcttcttagtcargtaccg^[^ ^39^ ^]^
	Clostridium leptum	gcacaagcagtggagt	cttcctccgtttgtcaa^[^ ^39^ ^]^
	Lactobacillus spp.	gaggcagcagtagggaatcttc	ggccagttactacctctatccttcttc^[^ ^39^ ^]^
Bacteroidetes	Bacteroides/Prevotella	tcctacgggaggcagcagt	caatcggagttcttcgtg^[^ ^39^ ^]^
Verrucomicrobia	Akkermansia muciniphila	cagcacgtgaaggtggggac	ccttgcggttggcttcagat^[^ ^40^ ^]^
**Housekeeping**			
Total Bacteria	Total bacteria	actcctacgggaggcag	gtattaccgcggctgctg^**[**^ ^**39**^ ^**]**^

Forward and reverse sequences for qPCR amplification in mouse caecal content.

### Statistical analysis

Data are presented as means ± SEM. Equality of variances between groups was assessed by Levene’s test. When homogeneity of variances was assumed, one-way ANOVA was used to determine the significance of the different parameters between groups. If there was a significant difference, a Bonferroni test was used to determine where the difference lay and to correct for multiple testing. When homogeneity of variances was not assumed, data were log transformed. Linear relationships between key variables were tested using Pearson's correlation coefficients. Threshold of significance was set at P<0.05. The analysis was performed using the SPSS program for Windows version 21.0 (SPSS, Chicago, IL, USA).

## Results

### CLA supplementation modulates the expression of regulatory proteins in the stomach

**Fig 1:**
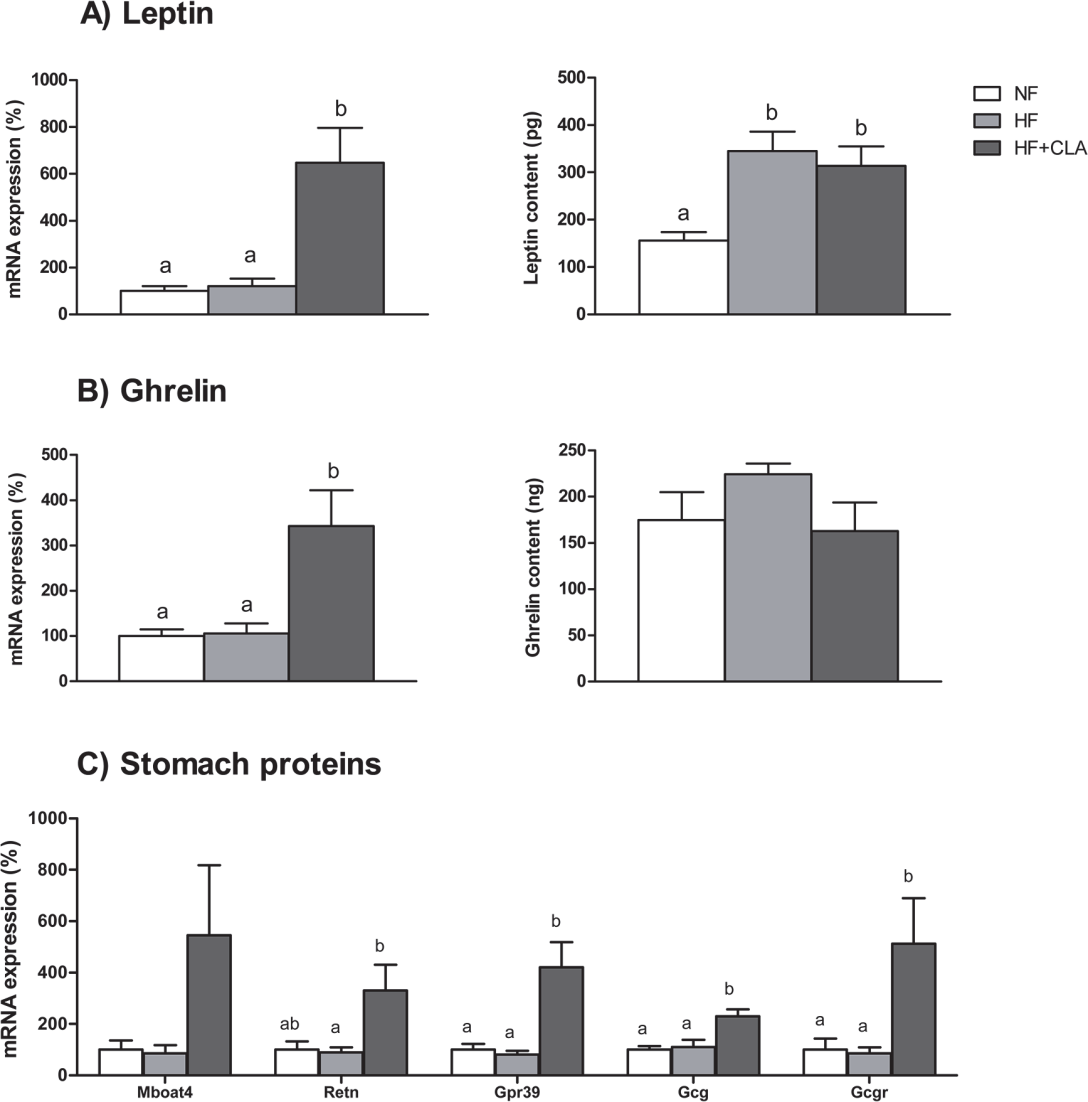
Effect of CLA supplementation on mRNA expression and protein levels of gastric proteins in mice. Expression of stomach proteins were analysed in mice after 54 days of supplementation with CLA. (A) Leptin mRNA (%) expression was increased by CLA, and protein content (pg) was higher in both HF and CLA groups. (B) Ghrelin mRNA (%) expression was also higher in CLA animals, whereas protein levels (ng) did not show significant differences amongst groups. (C) Gastric resistin (*Retn*), G protein-coupled receptor 39 (*Gpr39*), glucagon (*Gcg*) and glucagon receptor (*Gcgr*) expression increased by CLA supplementation, whereas ghrelin o-acyltransferase (*Mboat4)* did not. Data are the mean ± SEM of 8 animals/group. Letters indicate differences amongst groups; one-way ANOVA followed by Bonferroni test (p<0.05).

Expression of proteins associated to energy metabolism and regulation of food intake was determined in mouse stomach. Leptin mRNA expression in the HF group was not altered, however leptin protein was increased in these animals (2-fold vs. NF, p = 0.003). On the other hand, supplementation with CLA caused a 6-fold higher expression of leptin mRNA (in comparison with NF, p = 0.001), which was in accordance with higher content of gastric leptin (2-fold, p = 0.014 vs. NF) (Fig 1A). CLA-fed animals also exhibited increased ghrelin mRNA expression (3-fold, p = 0.006 vs.NF), but no changes were seen amongst groups regarding ghrelin protein (Fig 1B).

Furthermore, CLA supplementation was associated to increased expression of all stomach proteins analysed and statistical significance was attained in the case of resistin (Retn) (3-fold, p = 0.026), G protein-coupled receptor 39 (*Gpr39)* (4-fold, p = 0.001), Glucagon (*Gcg)* (2-fold, 0.001) and Glucagon Receptor (*Gcgr)* (5-fold, p = 0.011) (Fig 1C).

### Caecum microbiota is modulated by CLA

**Fig 2:**
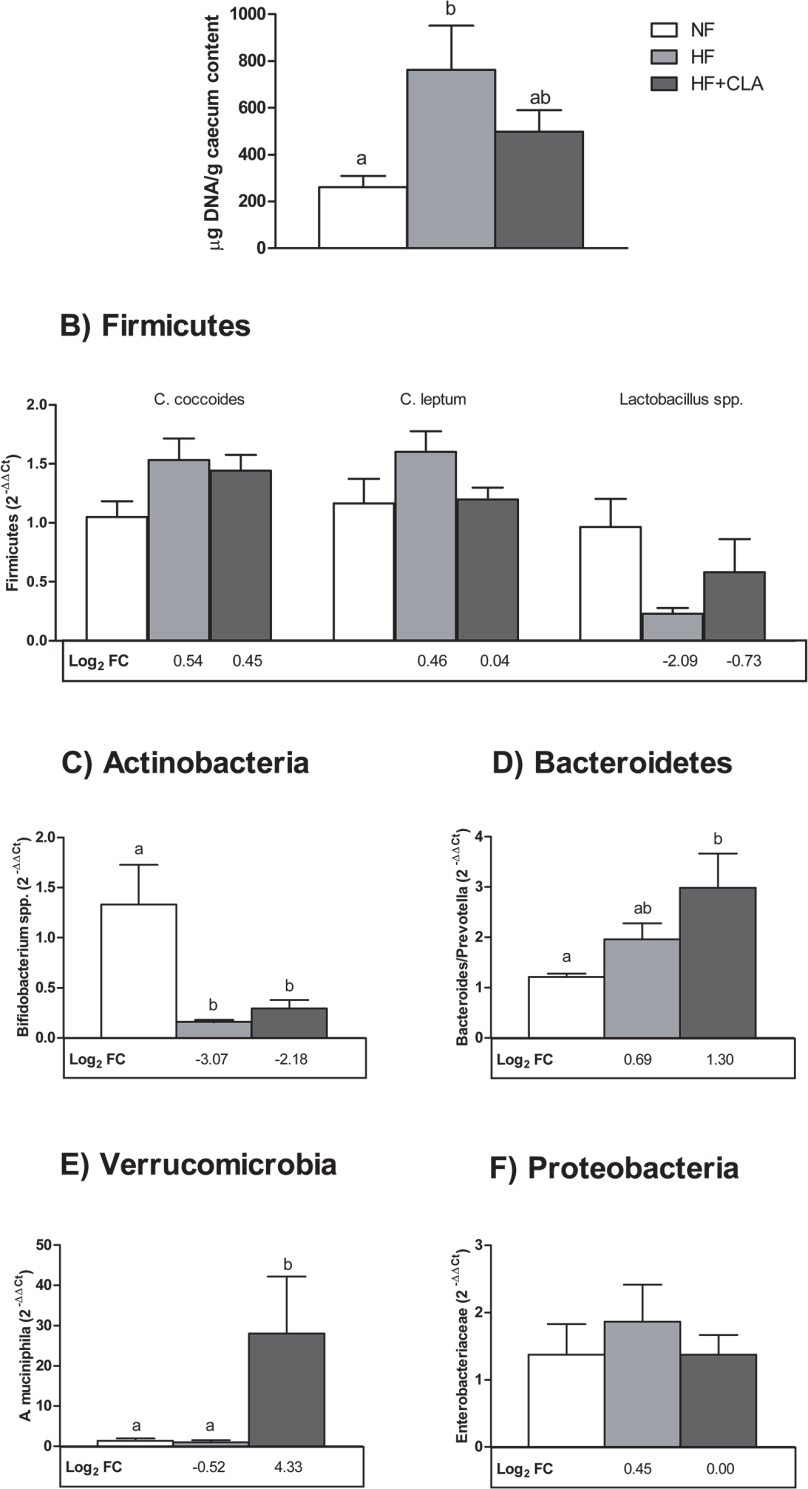
DNA levels of representative bacterial species in mice caecum are altered by diet. Bacterial species from caecum content was analysed in all groups. (A) Bacterial DNA levels in caecum content (μg bacterial DNA/g caecum content) was increased significantly by HF diet. DNA abundance of 16S rRNA gene of representative bacterial species (B) Firmicutes, (C) Actinobacteria, (D) Bacteroidetes, (E) Verrucomicrobia and (F) Proteobacteria was analysed in mouse caecum and normalised with the average of the NF group (2^−ΔΔCt^). Fold change respect to NF group was calculated (Log_2_ FC) and is indicated below each column. Data are mean ± SEM of 6–8 animals/group. Letters indicate differences amongst groups; one-way ANOVA followed by Bonferroni test (p<0.05). When homogeneity of variances was not assumed, data were log transformed.

To further look into the effect of CLA, caecum content of mice was analysed in order to determine bacterial species potentially associated to obesity and energy metabolism. A significant increase in bacterial DNA caecum content was observed in mice fed a HF diet (3-fold vs. NF, p = 0.032), which decreased with CLA supplementation and showed no differences compared to NF animals (Fig 2A). This was accompanied by differences in gut microbiota composition.

No statistically significant changes were found concerning the Firmicutes content under the different dietary treatments (Fig 2B). HF feeding was mainly associated to a decrease in *Bifidobacterium* spp. (p = 0.009 vs. NF; with a fold change (Log_2_ FC) of -3.07). CLA supplementation did not counteract this reduction, presenting similar values (p = 0.034 vs. NF; -2.18 Log_2_ FC) (Fig 2C). In contrast, animals receiving CLA showed a significant increase in two bacterial species of interest: *Bacteroides/Prevotella* (p = 0.021 vs. NF; 1.30 Log_2_ FC) (Fig 2D) and *A*. *muciniphila*, which dramatically increased compared to both NF (p = 0.014; 4.33 Log_2_ FC) and HF (p = 0.002) (Fig 2E). No significant changes on *Enterobacteriaceae* profile were seen (Fig 2F).

### Caecum microbiota correlates with body weight and body fat

We next tested the hypothesis that abundance of specific bacterial species in mouse caecum contents could be associated to modulation of body weight and body fat. On one hand, *C*. *coccoides* (r = .433, p = 0.044) and *C*. *leptum* (r = .488, p = 0.021), both belonging to the Firmicutes’ group, showed positive correlations with body weight. *Bacteroides/Prevotella* also showed a positive correlation (r = .581, p = 0.006) with body weight. On the other, body fat was negatively correlated with *Bifidobacterium* spp. (r = -.547, p = 0.008). The correlation matrix is presented in Table 3.

**Table 3:** Correlations between bacterial species in caecum contents with body weight and body fat of mice.

Bacterial Species	Body Weight		Body Fat		
	R	P	R	P
*Clostridium coccoides*	.433*	0.044	.415	0.055
*Clostridium leptum*	.488*	0.021	.165	0.464
*Bacteroidetes*	.581**	0.006	.203	0.378
*Bifidobacterium* spp.	-.407	0.060	-.547**	0.008

Linear relationships were tested using Pearson’s correlation coefficients (R). Significant correlations are marked as follows:

* = p<0.05,

** = p<0.01.

## Discussion

The present study provides evidence that CLA supplementation under a HF diet has a noticeable effect on particular sites of the gastrointestinal tract in mouse, by increasing gastric protein expression and by promoting a prebiotic effect on gut microbiota. Considering that the gastrointestinal tract integrates the interplay of food, microbiota and metabolic effects on the host, these results may be relevant for the development of weight management strategies, since CLA are compounds used for the reduction of fat mass in humans [10–13].

Gut hormones are secreted in the stomach in response to food intake and play a key role in signalling food intake to the brain [41]. Leptin and ghrelin constitute two of the most studied proteins involved in energy metabolism, both being secreted in relevant amount by the gastric mucosa [42–47]. In accordance with leptin mRNA levels, higher gastric protein content was also observed, which is in accordance with the increased plasma leptin levels described in these animals [35]. It has been previously described that high-fat feeding stimulates the gastric leptin signalling pathway [44], an effect which would not be counteracted by CLA and would contribute to partially explain why no differences were seen regarding food intake in this experimental set up [35]. On the other hand, ghrelin gene is a gut orexigenic hormone which is mainly regulated by feeding [48], and although mRNA expression increased with CLA, protein ghrelin levels were not changed. The discrepancies observed between mRNA expression and protein levels would be associated to the presence of diurnal rhythms described for both ghrelin and leptin in the gastric environment which allow for a better metabolic control[49]. In addition to these main gastric proteins, we analysed ghrelin o-acyltransferase (*Mboat4)*, resistin and *Gpr39*, as well as glucagon, somatostatin and their receptors, proteins which are also involved in energy balance and have been described in the gastric mucosa [50–56], although their potential in weight management has not been thoroughly studied. In this context, *Mboat4* was of particular interest as it is the enzyme responsible for the acylation of ghrelin [56] and is activated by dietary lipids which act as acylation substrates [57]. Interestingly, all of the proteins analysed showed increased expression with CLA supplementation, suggesting that CLA isomers are specifically sensed by the genes encoding gastric proteins which respond to food cues.

Additionally to the abovementioned effects, CLA promoted changes in the gut microbiota of the lower part of the gastrointestinal tract. In recent years, it has been proposed that in obese states there is an increased ratio of Firmicutes to Bacteroidetes as well as a loss of bacterial diversity [26–28] although currently more focus is being put on bacterial species for host metabolism characterization as new data emerge (discussed in a recent review [58]). Neither CLA supplementation nor HF diet alone was associated with changes amongst bacterial species in the Firmicutes’ phylum. However, HF feeding lowered *Bifidobacterium* spp., in accordance with previous animal studies [59–62], a reduction which was not counteracted by CLA. Considering supplementation was not able to re-establish normal body weight [35], a positive association with body weight was found for both *C*. *coccoides* and *C*. *leptum*, in agreement with the potential adverse role of these bacterial species on obesity [33, 63, 64], as well as a negative correlation with *Bifidobacterium* spp., an association which has also been previously found in both animal models and human studies [60–63].

In contrast, CLA induced a prebiotic effect in supplemented animals. *Bacteroides/Prevotella* is a bacterial species known to use dietary polysaccharides in a prebiotic fashion [65]. A remarkable increase was found under CLA supplementation suggesting that CLA was able to confer a prebiotic effect. This is supported by a very high induction of *A*. *muciniphila* growth by CLA. The presence of this mucin-degrading bacterial species, which resides in the mucus layer, is associated to a healthy mucosa and is generally reduced in obese states [66–68]. Everard et al. [69] have recently demonstrated that oligofructose restores *A*. *muciniphila* content in obese animals, and this is associated with an improvement of their metabolic profile. Therefore, the increased caecal content of *A*. *muciniphila* found under CLA supplementation suggests that this compound was exerting a prebiotic action on this bacterial species too. To our knowledge, this is the first evidence of a CLA-prebiotic effect favouring the specific growth of potentially beneficial bacterial species in the gut. However, this increase in both *Bacteroides/Prevotella* and *A*. *muciniphila* was not enough to allow a full recovery since these animals remain obese [35]. We cannot rule out that a higher CLA dose and/or longer treatment, which is generally associated with a leaner phenotype [70], would cause an even higher increase in *A*. *muciniphila* content and have more significant effects on metabolic parameters. This would fit with the lower induction of *A*. *muciniphila* found in the present study in comparison with others [69, 71].

Overall, our data show that gastrointestinal tract is a first site for action of the bioactive isomers of CLA, able to modulate gastric responses as well as microbiota-host metabolism. We cannot rule out the potential interplay between gastric environment and bacterial growth associated to food cues accompanying CLA intake. However, CLA induced the expression of genes encoding gastric proteins related with regulation of energy balance and exerted a prebiotic effect on selected bacterial species. Growth of potentially beneficial bacterial species, specifically *Bacteroidetes/Prevotella* and *A*. *muciniphila*, suggests CLA confers a prebiotic effect, which could contribute to a healthier metabolic profile. Further and thorough research of how specific dietary treatments influence specific physiological components, such as the gastrointestinal tract, will help elucidate their impact on specific conditions such as obesity, and develop efficient body weight management strategies.

## Supporting Information

S1 Table Detailed composition of diets. Composition of normal and high fat diets used throughout the experiment.
(DOCX)
