# Supplementary material for: Conjugated Linoleic Acid Supplementation under a High-Fat Diet Modulates Stomach Protein Expression and Intestinal Microbiota in Adult Mice
Source: PLoS One. 2015 Apr 27;10(4):e0125091. doi: 10.1371/journal.pone.0125091 (PMC4411160; doi:10.1371/journal.pone.0125091)
Supplement: S1 Table — Composition of normal and high fat diets used throughout the experiment. (DOCX) [file pone.0125091.s001.docx]

|  | **Normal Fat** | **High Fat** |
| --- | --- | --- |
|  | g/kg | |
| **Fat** |  |  |
| Soybean Oil | 32 | 66 |
| Lard | 20 | 120 |
| *t-*ButylHydroquinone | 0.014 | 0.014 |
| **Protein** |  |  |
| Casein | 214 | 212 |
| DL-Methyonine | 3 | 3 |
| **Carbohydrate** |  |  |
| Sucrose | 400 | 350 |
| Corn Starch | 250 | 0 |
| **Cellulose** | 50 | 50 |
| **Mineral Mix** | 7 | 7 |
| **Calcium Carbonate** | 10 | 10 |
| **Potassium Phosphate Monobasic** | 8 | 8 |
| **Potassium Citrate Monohydrate** | 1.6 | 1.6 |
| **Vitamin Mix V10037** | 10 | 10 |
| **Choline Bitartrate** | 2.5 | 2.5 |
| **Fat, kJ%** | 12.1 | 43.2 |
| **Protein, kJ%** | 19.8 | 19.6 |
| **Carbohydrate, kJ%** | 68.2 | 37.2 |
